# Antidepressant effects of combination of brexpiprazole and fluoxetine on depression-like behavior and dendritic changes in mice after inflammation

**DOI:** 10.1007/s00213-016-4483-7

**Published:** 2016-11-15

**Authors:** Min Ma, Qian Ren, Chun Yang, Ji-chun Zhang, Wei Yao, Chao Dong, Yuta Ohgi, Takashi Futamura, Kenji Hashimoto

**Affiliations:** 1 Division of Clinical Neuroscience, Chiba University Center for Forensic Mental Health, 1-8-1 Inohana, Chiba, 260-8670 Japan; 2 Department of CNS Research, New Drug Research Division, Otsuka Pharmaceutical Co., Ltd., Tokushima, Japan

**Keywords:** Brexpiprazole, Fluoxetine, Inflammation, Spine, SSRI

## Abstract

**Rationale:**

Addition of low doses of atypical antipsychotic drugs with selective serotonin reuptake inhibitors (SSRIs) could promote a rapid antidepressant effect in treatment-resistant patients with major depression. Brexpiprazole, a new atypical antipsychotic drug, has been used as adjunctive therapy for the treatment of major depression.

**Objectives:**

The present study was undertaken to examine whether brexpiprazole could augment antidepressant effects of the SSRI fluoxetine in an inflammation model of depression.

**Methods:**

We examined the effects of fluoxetine (10 mg/kg), brexpiprazole (0.1 mg/kg), or the combination of the two drugs on depression-like behavior, alterations in the brain-derived neurotrophic factor (BDNF) - TrkB signaling, and dendritic spine density in selected brain regions after administration of lipopolysaccharide (LPS) (0.5 mg/kg).

**Results:**

Combination of brexpiprazole and fluoxetine promoted a rapid antidepressant effect in inflammation model although brexpipazole or fluoxetine alone did not show antidepressant effect. Furthermore, the combination significantly improved LPS-induced alterations in the BDNF - TrkB signaling and dendritic spine density in the prefrontal cortex, CA3 and dentate gyrus, and nucleus accumbens.

**Conclusions:**

These results suggest that add-on of brexpiprazole to fluoxetine can produce a rapid antidepressant effect in the LPS inflammation model of depression, indicating that adjunctive therapy of brexpiprazole to SSRIs could produce a rapid antidepressant effect in depressed patients with inflammation.

## Introduction

Accumulating evidence suggests that inflammation plays a role in the pathophysiology of major depressive disorder (MDD) (Dantzer et al. 2008; Hashimoto 2015b; Miller and Raison 2015; Strawbridge et al. 2015). A meta-analysis shows higher blood levels of pro-inflammatory cytokines in drug-free depressed patients, compared with healthy controls (Dowlati et al. 2010). Peripheral administration of the bacterial endotoxin lipopolysaccharide (LPS) induces depression-like behavior in rodents after the induction of inflammation (Dantzer et al. 2008; O’Connor et al. 2009; Remus and Dantzer 2016; Zhang et al. 2016). LPS-induced depression-like behavior can be blocked by pretreatment with antidepressants, including selective serotonin reuptake inhibitors (SSRIs) and serotonin-norepinephrine reuptake inhibitors (SNRIs) (Dong et al. 2016; Ma et al. 2014; Ohgi et al. 2013; Yao et al. 2015). These findings suggest that inflammation might be associated with depressive symptoms.

Several clinical studies demonstrate that addition of low doses of atypical antipsychotic drugs (e.g., aripiprazole, olanzapine, quetiapine, risperidone, ziprasidone) to SSRIs to rapidly enhance the antidepressant effects in depressed patients, including treatment-resistant patients (Barbee et al. 2004; Brunner et al. 2014; Nelson and Papakostas 2009; Ozaki et al. 2015; Papakostas et al. 2005, 2007, 2015; Rogóz 2013; Shelton and Papakostas 2008). Brexpiprazole (7-{4-[4-(1-benzothiophen-4-yl)piperazin-1-yl]butoxy}quinolin-2(1H)-one), a serotonin-dopamine activity modulator, binds with high affinity (Ki < 1 nM) to human serotonin (5-HT) 5-HT_1A_, 5-HT_2A_-, dopamine D_2_ (D_2L_)- and adrenergic α_1B_-, α_2C_-receptors. It displays partial agonism at 5-HT_1A_ and D_2_ receptors, and potent antagonism of 5-HT_2A_ receptors and α_1B/2C_–adrenoceptors (Maeda et al. 2014a). Furthermore, brexpiprazole could potentiate nerve growth factor (NGF)-induced neurite outgrowth in PC12 cells via 5-HT_1A_ and 5-HT_2A_ receptors (Ishima et al. 2015). Moreover, brexpiprazole showed antipsychotic-like and procognitive effects in rodents (Maeda et al. 2014b; Yoshimi et al. 2014, 2015). Brexpiprazole has shown efficacy as adjunctive treatment of MDD (Citrome 2015; McKeage 2016; Stahl 2016; Thase et al. 2015a, 2015b).

The purpose of this study is to examine whether combination of brexpiprazole and SSRI fluoxetine could improve depression-like behaviors and alterations in the brain-derived neurotrophic factor (BDNF) - TrkB signaling and dendritic spine density in the selected brain regions after a single LPS administration.

## Material and methods

### Animals

Male adult C57BL/6 mice (8 weeks old) weighing 20–25 g were purchased from SLC Japan (Hamamatsu, Shizuoka, Japan). The mice were housed in clear polycarbonate cages (22.5 × 33.8 × 14.0 cm) in groups of 4 or 5 individuals under a controlled 12/12-h light–dark cycle (light from 7:00 a.m to 7:00 p.m.), with the room temperature kept at 23 °C ± 1 °C and humidity at 55% ± 5%. The mice were given free access to water and food pellets specifically designed for mice. The experimental procedure was approved by the Animal Care and Use Committee of Chiba University Graduate School of Medicine.

### Drugs and drug administration

LPS (0.5 mg/kg; L-4130, serotype 0111:B4, Sigma-Aldrich, St Louis, MO, USA) was dissolved in distilled water. Saline (10 ml/kg) or LPS (0.5 mg/kg) was administered intraperitoneally (i.p.). Brexpiprazole was synthesized at Otsuka Pharmaceutical Co., Ltd. (Tokyo, Japan). Vehicle (0.5% CMC; 10 ml/kg), fluoxetine (10 mg/kg, Wako Chemical Co., Ltd., Tokyo, Japan), brexpiprazole (0.1 mg/kg), or fluoxetine (10 mg/kg) plus brexpiprazole (0.1 mg/kg) were administered orally. The doses of brexpiprazole (0.1 mg/kg) and fluoxetine (10 mg/kg) were selected as reported previously (Maeda et al. 2014a, 2014b; Rogóz 2013; Yoshimi et al. 2015). The time schedule of behavioral tests after oral administration of drugs was selected as previously reported (Hirano et al. 2005). Other chemicals were purchased from commercial sources.

### Behavioral tests

Behavioral tests were performed as previously reported (Ren et al. 2015, 2016; Yang et al. 2015b; Zhang et al. 2015a, 2015b).

#### Locomotion

Mice were placed in experimental cages (L560 × W560 × H330 mm), and locomotor activity was counted by the SCANET MV-40 (MELQUEST, Toyama, Japan). The cumulative exercise was recorded for 60  min. All cages were cleaned between testing session.

#### Tail suspension test

A small piece of adhesive tape was placed at 2 cm from the tip of the tail and punched with a single hole that serves to hang the mice on a hook. The immobility time of each mouse was recorded for 10 min. Mice were considered immobile only when they hung passively and completely motionless.

#### Forced swim test

Animals were tested in an automated forced-swim apparatus using SCANET MV-40 (MELQUEST Co., Ltd., Toyama, Japan). The mice were placed individually in a cylinder (Diameter 23 cm; Height 21 cm), containing 15 cm of 23 ± 1 °C warm water. Immobility time was calculated by subtracting active time from total time, using the apparatus analysis software. Cumulative immobility time was scored for 6  min during the test. The TST and FST were performed 2 and 4 h after the LMT, respectively.

### Western blot analysis of BDNF, and its precursor proBDNF, TrkB, and phosphorylated-TrkB

Western blot analysis was performed as reported previously (Ren et al. 2015, 2016; Yang et al. 2015b; Zhang et al. 2015a, 2015b). Mice were killed by cervical dislocation and brains were rapidly removed from the skull. Approximately 1 mm thick coronal sections were cut and bilateral tissue punches of prefrontal cortex (PFC), nucleus accumbens (NAc), striatum, CA1, CA3, and dentate gyrus (DG) of the hippocampus were dissected on ice using a SZ-LED Kenis light microscope (Osaka, Japan), and stored at −80 °C. Tissue samples were homogenized in Laemmli lysis buffer. Aliquots (20 μg) of protein were measured using the DC protein assay kit (Bio-Rad), and incubated for 5 min at 95 °C, with an equal volume of 125 mM Tris-HCl, pH 6.8, 20% glycerol, 0.1% bromophenol blue, 10% β-mercaptoethanol, 4% SDS, and subjected to SDS polyacrylamide gel electrophoresis using AnyKD minigels (Mini-PROTEAN TGX Precast Gel; BioRad). Proteins were transferred onto PVDF membranes using a Trans Blot Mini Cell (Bio-Rad). For immunodetection, the blots were blocked with 2% BSA in TBST (TBS + 0.1% Tween-20) for 1 h at room temperature, and kept with primary antibodies overnight at 4 °C. The following primary antibodies were used: BDNF (1:200; H-117, Cat#: sc-20981, Santa Cruz Biotechnology), phosphor-TrkB (Tyr-706) (1:200; Cat#: sc135645, Santa Cruz Biotechnology), TrkB (80E3) (1:1000; Cat#: 4603, Cell Signaling Technology). The next day, blots were washed three times in TBST and incubated with horseradish peroxidase-conjugated anti-rabbit antibody (1:10,000) 1 h at room temperature. After a final three washes with TBST, bands were detected using enhanced chemiluminescence (ECL) plus the Western Blotting Detection system (GE Healthcare Bioscience). The blots were then washed three times in TBST and incubated with the primary antibody directed against β-actin (1:10,000; Sigma-Aldrich). Images were captured with a Fuji LAS3000-mini imaging system (Fujifilm, Tokyo, Japan), and immunoreactive bands were quantified.

### Golgi staining

Golgi staining was performed using the FD Rapid GolgiStain™ Kit (FD Neuro Technologies, Inc., Columbia, MD), following the manufacturer’s instructions (Zhang et al. 2015a; Ren et al. 2015). Two hours after oral administration of vehicle (10 ml/kg), fluoxetine (10 mg/kg), brexpiprazole (0.1 mg/kg), or fluoxetine (10 mg/kg) plus brexpiprazole (0.1 mg/kg), animals were deeply anesthetized with sodium pentobarbital, and brains were removed from the skull and rinsed in double distilled water. Brains were immersed in the impregnation solution, made by mixing equal volumes of Solution A and B, overnight and then stored in fresh solution, for 2 weeks in the dark. Brains were transferred into Solution C overnight and then stored in fresh solution at 4 °C for 1 week, in the dark. Coronal brain sections (100 μm thickness) were cut on a cryostat (3050S, Leica Microsystems AG, Wetzlar, Germany), with the chamber temperature set at −20 °C. Each section was mounted in Solution C, on saline-coated microscope slides. After absorption of excess solution, sections were dried naturally, at room temperature. Dried sections were processed following the manufacturer’s instructions. Briefly, images of dendrites within CA1, CA3, and DG of the hippocampus, prelimbic (PrL) and inflalimbic (IL) areas of medial PFC (mPFC), and shell and core of NAc were captured using a 100× objective with a Keyence BZ-9000 GenerationIImicroscope (Osaka, Japan). Spines were counted along CA1, CA3, DG, PrL and IL of mPFC, and shell and core of NAc dendrites starting from their point of origin from the primary dendrite, as previously reported (Zhang et al. 2015a; Ren et al. 2015). For spine density measurements, all clearly evaluable areas containing 50–100 μm of secondary dendrites from each imaged neuron were used. To determine relative spine density, spines on multiple dendritic branches from a single neuron were counted to obtain an average spine number per 10 μm. For spine number measurements, only spines that emerged perpendicular to the dendritic shaft were counted. Three neurons per section, three sections per animal, and six animals were analyzed. The average value for each region, in each individual, was obtained. These individual averages were then combined to yield a grand average for each region.

### Statistical analysis

The data show as the mean ± standard error of the mean (S.E.M.). Analysis was performed using PASW Statistics 20 (formerly SPSS Statistics; Tokyo, Japan). Comparisons between groups were performed using the one-way analysis of variance (ANOVA), followed by post hoc Fisher’s least significant difference (LSD) tests. The *P* values of less than 0.05 were considered statistically significant.

## Result

### Effects of fluoxetine and brexpiprazole on depression-like behavior in mice after LPS administration

**Fig. 1:**
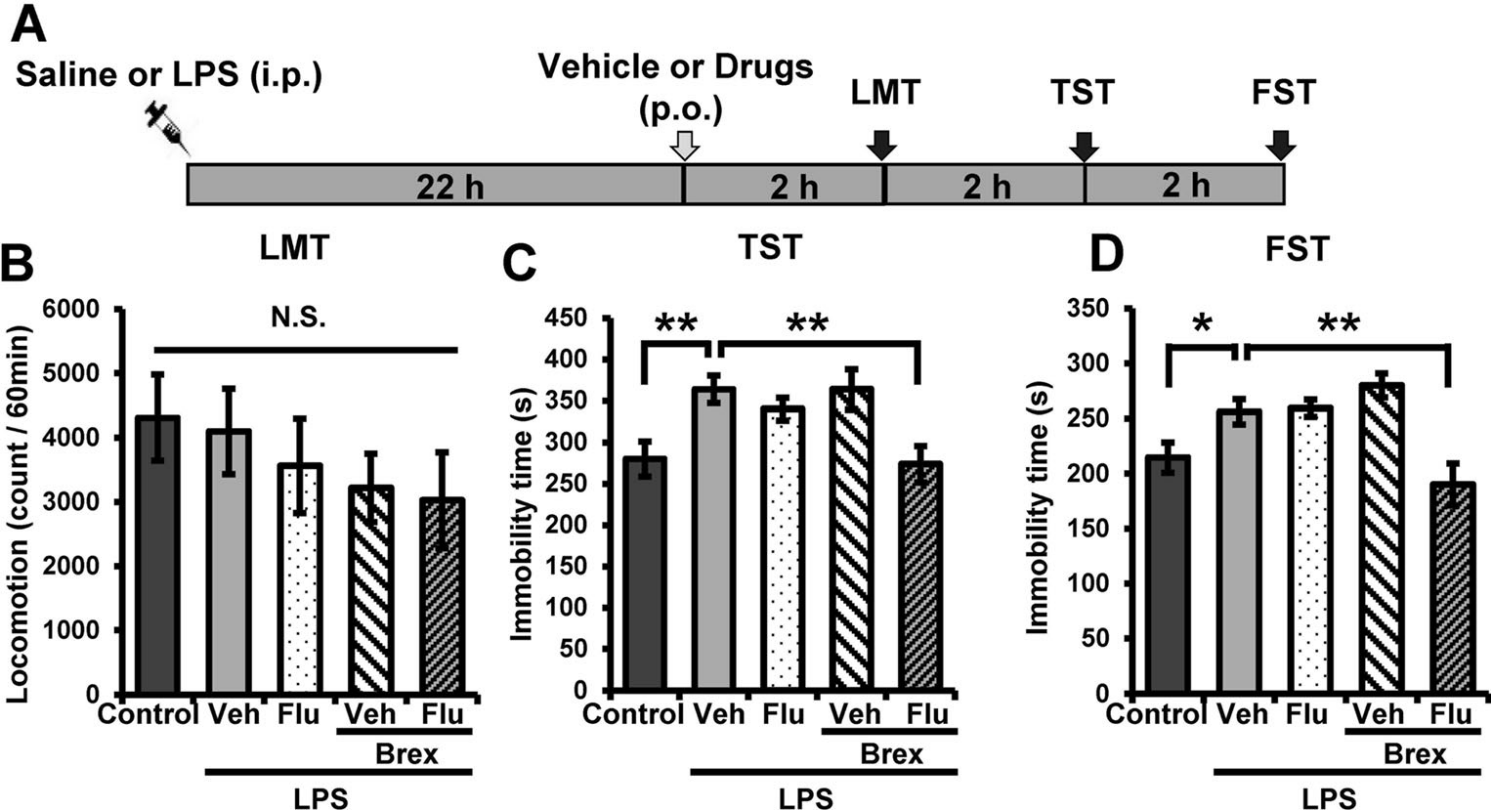
Antidepressant effects of combination of brexpiprazole and fluoxetine in inflammation model **a** Schedule of treatment and behavioral tests. Saline (10 ml/kg) or LPS (0.5 mg/kg) was administered i.p. Vehicle (10 ml/kg), fluoxetine (10 mg/kg), brexpiprazole (0.1 mg/kg), or fluoxetine (10 mg/kg) plus brexpiprazole (0.1 mg/kg) were administered orally 22 h after LPS administration. Locomotion (LST), tail-suspension test (TST), and forced swim test (FST) were performed 2, 4, and 6 h after oral administration. **b** LMT. **c** TST. **d** FST. Data are shown as mean ± S.E.M. (*n* = 7–9). *P < 0.05, **P < 0.01, ***P < 0.001 compared to vehicle-treated LPS group (one-way ANOVA, followed post hoc LSD test). *N.S*. not significant, *Veh* vehicle, *Flu* fluoxetine, *Brex* brexpiprazole

Vehicle, fluoxetine (10 mg/kg), brexpiprazole (0.1 mg/kg), or fluoxetine (10 mg/kg) plus brexpiprazole (0.1 mg/kg) were administered orally into mice 22 h after LPS (0.5 mg/kg) administration (Fig. 1a). In the locomotion test (LMT), there were no differences (*F*_4,38_ = 0.819, *P* = 0.522) among the five groups (Fig. 1b). One-way ANOVA of TST and FST data revealed the statistical results (TST (Fig. 1c)): *F*_4,38_ = 4.922, *P* = 0.003, FST ((Fig. 1d): *F*_4,38_ = 7.346, *P* < 0.0001). In the TST and FST, combination of fluoxetine and brexpiprazole significantly attenuated the increased immobility time in mice after LPS administration (Fig. 1c, d). In contrast, fluoxetine or brexpiprazole alone did not alter the increased immobility time for TST and FST after LPS administration (Fig. 1c, d). These findings suggest that adjunctive treatment of brexpiprazole with fluoxetine showed a rapid antidepressant effect on LPS-induced depression model.

### Effects of fluoxetine and brexpiprazole on BDNF-TrkB signaling in selected brain regions of mice after LPS administration

**Fig. 2:**
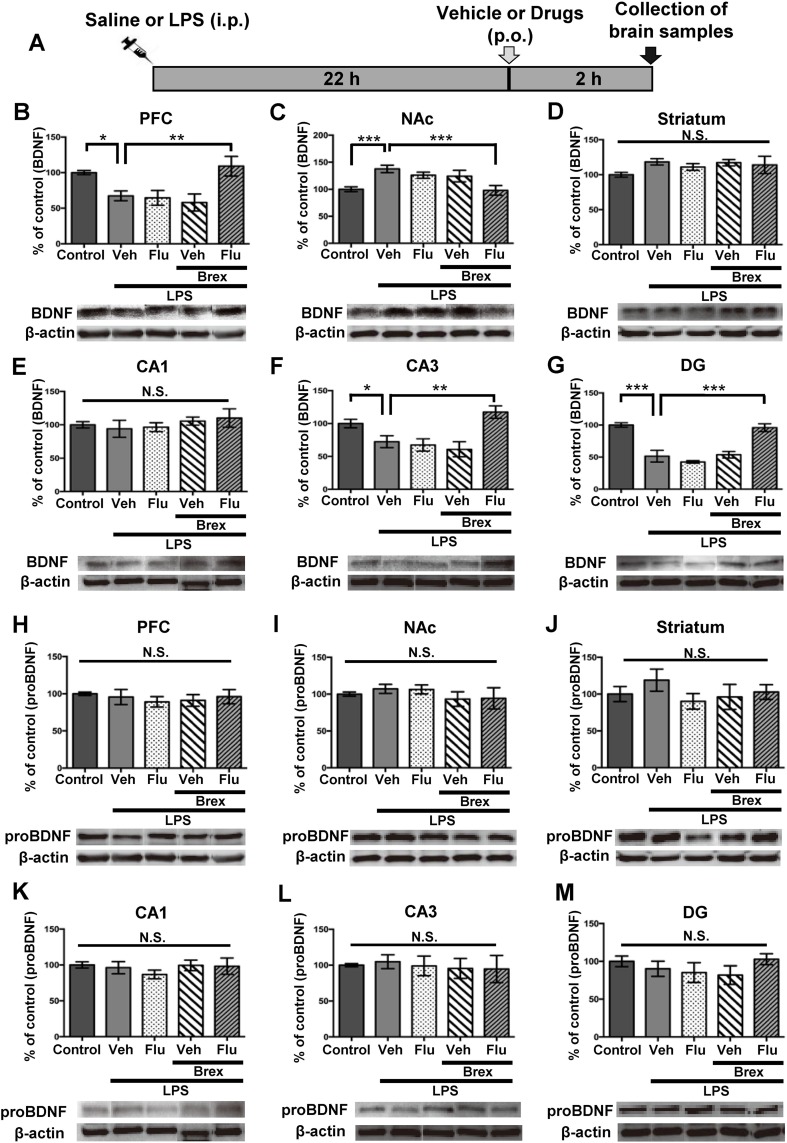
Effects of brexpiprazole and fluoxetine combination on the alterations in the BDNF and proBDNF in the brain regions after LPS administration **a** Schedule of treatment and collection of brain samples. Vehicle (10 ml/kg), fluoxetine (10 mg/kg), brexpiprazole (0.1 mg/kg), or fluoxetine (10 mg/kg) plus brexpiprazole (0.1 mg/kg) were administered orally 22 h after saline or LPS (0.5 mg/kg) administration. Brain regions were collected 2 h after administration of drugs. Western blot analysis of proBDNF (**b**–**g**), BDNF (mature form)(**h**–**m**), and β-actin in the brain regions (PFC, NAc, striatum, CA1, CA3, DG) was performed. **b**, **h** PFC. **c**, **i** NAc. **d**, **j** striatum. **e, k** CA1. **f**, **i** CA3. **g**, **m** DG. The values are expressed as a percentage of that of control mice. Representative data of Western blot analyses of proBDNF, BDNF, and β-actin in the mouse brain regions. Data are shown as mean ± S.E.M. (*n* = 6–8). *P < 0.05, **P < 0.01, ***P < 0.001 compared to vehicle-treated LPS group (one-way ANOVA, followed post hoc LSD test). *N.S*. not significant, *Veh* vehicle, *Flu* fluoxetine, *Brex* brexpiprazole

Since PFC, NAc, striatum, CA1, CA3 and DG of the hippocampus play a role in the depression-like phenotype in rodents (Ren et al. 2015; Shirayama et al. 2015; Yang et al. 2015a, 2015b; Zhang et al. 2015a, 2015b), we performed Western blot analysis of BDNF (mature form), its precursor proBDNF, TrkB, and phosphorylated TrkB (p-TrkB) in selected brain regions (PFC, NAc, striatum, DG, CA1, and CA3). Vehicle, fluoxetine (10 mg/kg), brexpiprazole (0.1 mg/kg), or fluoxetine (10 mg/kg) plus brexpiprazole (0.1 mg/kg) was administered orally into mice 22 h after LPS administration (Fig. 2a). Brain regions were collected 2 h after oral administration (Fig. 2a). One-way ANOVA of BDNF data revealed the statistical results (PFC: *F*_4,31_ = 5.785, *P* = 0.0013, NAc: *F*_4,33_ = 5.896, *P* = 0.0011, striatum: *F*_4,25_ = 1.165, *P* = 0.35, CA1: *F*_4,27_ = 0.501, *P* = 0.736; CA3: *F*_4,30_ = 7.265, *P* = 0.0003; DG: *F*_4,32_ = 17.24, *P* < 0.0001) (Fig. 2b–g). Combination of brexpiprazole and fluoxetine significantly attenuated decreased BDNF levels in the PFC, CA3, and DG regions after LPS administration (Fig. 2b, f, g). Furthermore, combination of brexpiprazole and fluoxetine significantly attenuated increased BDNF levels in the NAc after LPS administration (Fig. 2c). However, no regional differences of proBDNF protein levels were observed among the five groups (Fig. 2h–m).

**Fig. 3:**
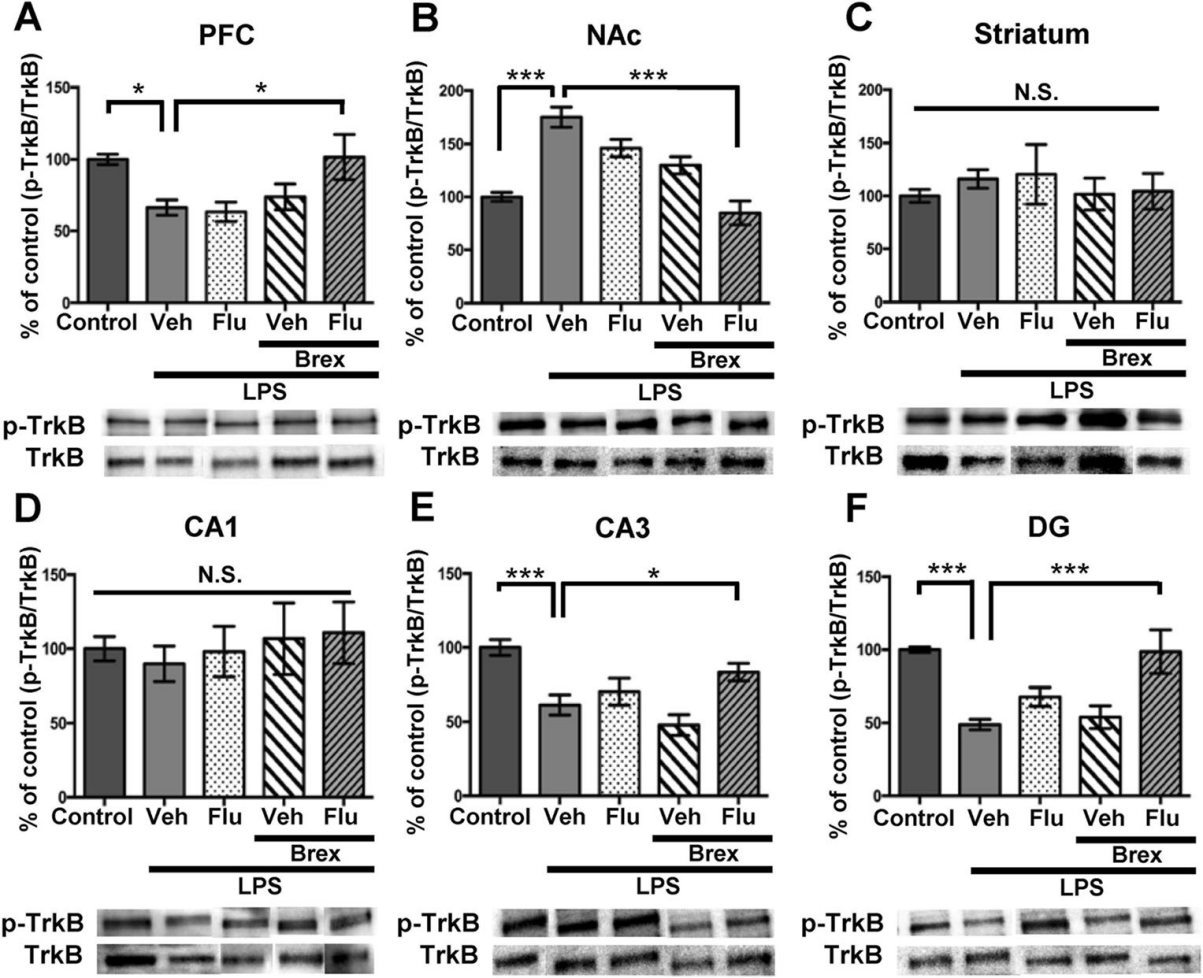
Effects of brexpiprazole and fluoxetine combination on the alterations in the phosphorylation of TrkB in the brain regions after LPS administration **a**–**f** The ratio of p-TrkB to total TrkB in the brain regions is shown. Representative data of Western blot analyses of p-TrkB and TRkB in the mouse brain regions. The values are expressed as a percentage of that of control mice. Representative data of Western blot analyses of BDNF and β-actin in the mouse brain regions. Data are shown as mean ± S.E.M. (*n* = 5–8). *P < 0.05, **P < 0.01, ***P < 0.001 compared to vehicle-treated LPS group (one-way ANOVA, followed post hoc LSD test). *N.S*. not significant, *Veh* vehicle, *Flu* fluoxetine, *Brex*: brexpiprazole

To clarify whether TrkB activation or inhibition underpins mechanistic action of brexpiprazole and fluoxetine combination, we performed Western blot analyses of TrkB and phosphorylated TrkB (p-TrkB), an activated form of TrkB, in samples from PFC, NAc, striatum, and CA1, CA3, DG of hippocampus. One-way ANOVA of p-TrkB/TrkB data revealed the statistical results (PFC: *F*_4,27_ = 3.179, *P* = 0.029, NAc: *F*_4,27_ = 17.67, *P* < 0.0001, striatum: *F*_4,30_ = 0.35, *P* = 0.842, CA1: *F*_4,30_ = 0.256, *P* = 0.904; CA3: *F*_4,26_ = 8.607, *P* = 0.0001; DG: *F*_4,25_ = 8.62, *P* = 0.0002) (Fig. 3a–f). Combination of brexpiprazole and fluoxetine significantly attenuated LPS-induced decrease of p-TrkB/TrkB ratio in the PFC, CA3, and DG regions (Fig. 3a, e, f). Furthermore, combination of brexpiprazole and fluoxetine significantly attenuated increased p-TrkB/TrkB ratio in the NAc after LPS administration (Fig. 3b). However, no regional differences of TrkB protein levels were observed among the five groups (data not shown).

### Effects of fluoxetine and brexpiprazole on alterations in the dendritic spine density in selected brain regions of mice after LPS administration

**Fig. 4.**
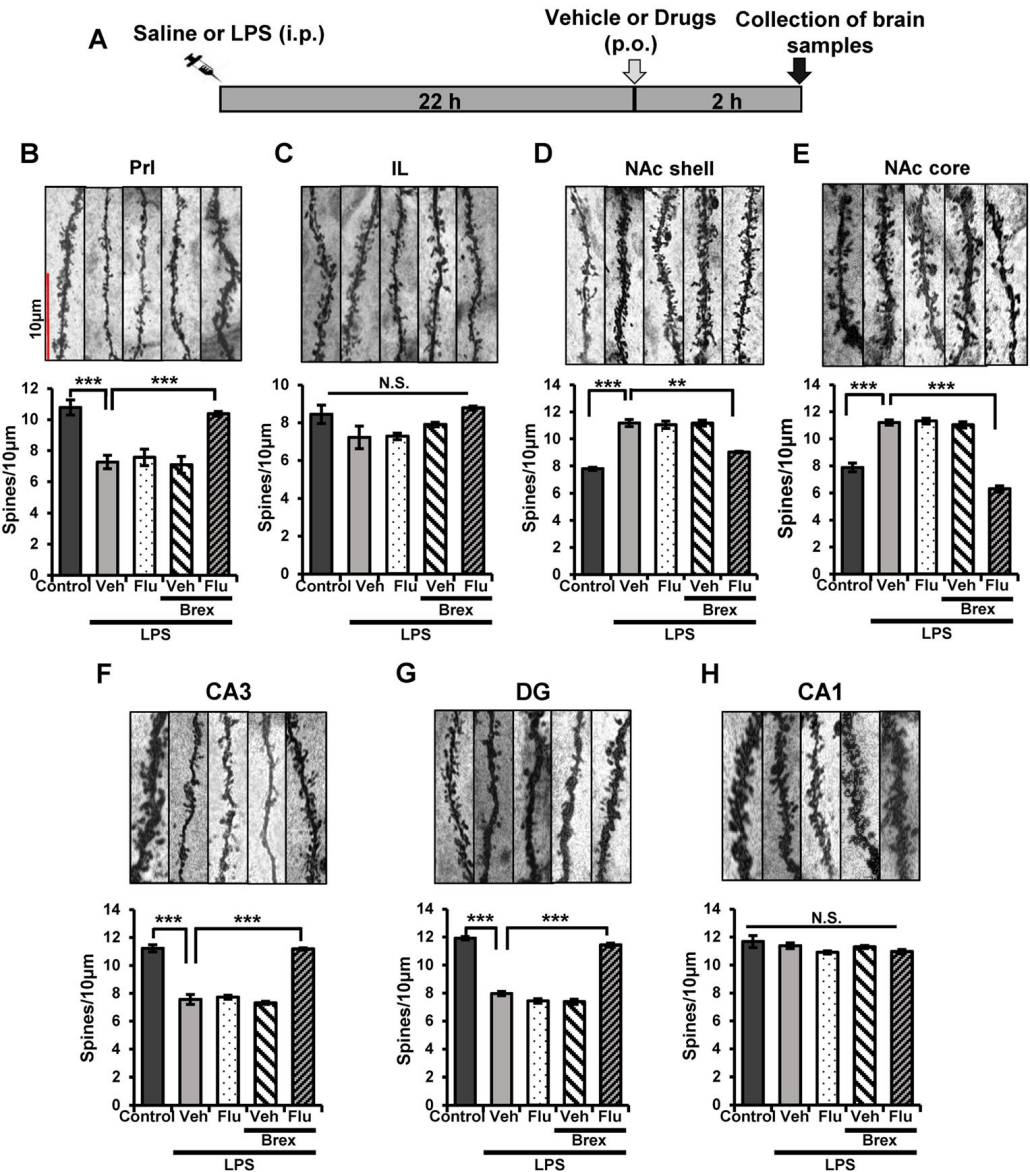
Effects of brexpiprazole and fluoxetine combination on the alterations in the dendritic spine density in the brain regions after LPS administration **a** Schedule of treatment, and collection of brain samples. Vehicle (10 ml/kg), fluoxetine (10 mg/kg), brexpiprazole (0.1 mg/kg), or fluoxetine (10 mg/kg) plus brexpiprazole (0.1 mg/kg) were administered orally 22 h after saline or LPS (0.5 mg/kg) administration. For Golgi staining, brain regions were collected 2 h after administration of drugs. **b**–**h** Golgi staining in the brain regions (PrL and IL regions of mPFC, core, and shell of NAc, CA1, CA3, and DG of hippocampus) was performed. Representative data of Golgi staining in the mouse brain regions. Data are shown as mean ± S.E.M. (*n* = 5–7). *P < 0.05, **P < 0.01, ***P < 0.001 compared to vehicle-treated LPS group (one-way ANOVA, followed post hoc LSD test*). N.S.* not significant, *Veh* vehicle, *Flu* fluoxetine, *Brex* brexpiprazole

A single administration of LPS (0.5 mg/kg) causes alterations in the dendritic spine density in the PFC, CA3, DG of hippocampus, and NAc (Zhang et al. 2015a). In this study, we examined whether combination of brexpiprazole and fluoxetine could affect alterations in the dendritic spine density in the prelimbic (PrL) and infralimbic (IL) regions of mPFC, shell and core of NAc, CA1, CA3, and DG of the hippocampus. Vehicle, fluoxetine (10 mg/kg), brexpiprazole (0.1 mg/kg), or fluoxetine (10 mg/kg) plus brexpiprazole (0.1 mg/kg) were administered orally into mice 22 h after LPS administration (Fig. 4a). Brain regions were collected 2 h after oral administration (Fig. 4a). One-way ANOVA of Golgi staining data revealed the statistical results (PrL of mPFC: *F*_4,24_ = 16.042, *P* < 0.0001, IL of mPFC: *F*_4,24_ = 1.236, *P* = 0.327, NAc core: *F*_4,24_ = 18.003, *P* < 0.0001, NAc shell: *F*_4,24_ = 12.501, *P* < 0.0001, CA1: *F*_4,26_ = 1.949, *P* = 0.138; CA3: *F*_4,26_ = 79.66, *P* < 0.0001; DG: *F*_4,26_ = 229.97, *P* < 0.0001)(Fig. 4b–h). Combination of brexpiprazole and fluoxetine significantly attenuated the LPS-induced decrease of spine density in the PrL of mPFC, CA3, and DG regions (Fig. 4b, f, g). Furthermore, combination of brexpiprazole and fluoxetine significantly attenuated LPS-induced increase of spine density in the core and shell of NAc (Fig. 4d, e). In contrast, administration of brexpiprazole or fluoxetine alone did not alter alterations in the dendritic spine density in these regions after LPS administration (Fig. 4b–h).

## Discussion

The major findings of this study are that combination of brexpiprazole and fluoxetine could promote a rapid antidepressant effect in an inflammation model of depression, although either drug alone did not show an antidepressant effect. Recently, we reported a rapid antidepressant effect of TrkB agonist 7,8-dihydroxyflavone (7,8-DHF) in the same model (Zhang et al. 2015a), indicating that the rapid antidepressant effect of combination of brexpiprazole and fluoxetine is similar to 7,8-DHF’s rapid antidepressant action. To the best of our knowledge, this is the first report showing a rapid antidepressant effect for combination of brexpiprazole and fluoxetine in inflammation model of depression. Therefore, it is likely that adjunction of brexpiprazole to SSRI therapy could promote a rapid antidepressant effect in depressed patients.

Studies using postmortem brain samples from depressed patients showed alterations in the BDNF expression in the hippocampus and NAc (Krishnan and Nestler 2008). In addition, serum levels of BDNF in depressed patients are lower than those of control subjects (Shimizu et al. 2003; Yoshida et al. 2012; Molendijk et al. 2014), suggesting that BDNF could be a biological marker for depression (Hashimoto 2010; 2015a). We previously reported a marked reduction of BDNF-TrkB signaling in the PFC, DG, and CA3, but not CA1, of inflammation model of depression (Zhang et al. 2015a). A single systemic administration of 7,8-DHF promoted a rapid antidepressant effect in inflammation model of depression (Zhang et al. 2015a), implicating BDNF-TrkB signal pathway in the PFC, DG, and CA3 in the antidepressant action of TrkB agonist. This is consistent with decreased BDNF protein levels in the PFC, DG, CA3, but not CA1, in inflammation model (Zhang et al. 2015a). In this study, we found that combination of brexpiprazole and fluoxetine could attenuate decreased BDNF-TrkB signaling in the PFC, CA3, and DG after inflammation. Therefore, it is possible that combination of brexpiprazole and fluoxetine might promote a rapid antidepressant effect by stimulation of BDNF-TrkB signaling in these regions.

Several studies have shown that NAc plays a critical role in depression (Nestler and Carlezon 2006; Shirayama et al. 2015; Zhang et al. 2015a, 2015b; Yang et al. 2015a). We also reported that inflammation, social defeat stress, and learned helplessness caused an increased BDNF-TrkB signaling within the NAc (Zhang et al. 2015a, 2015b; Shirayama et al. 2015; Yang et al. 2015a, 2015b). Taken together, this indicates that inflammation decreases BDNF in the hippocampus and PFC, but increases BDNF in the NAc, resulting in depression-like behavior in rodents. Interestingly, we found that combination of brexpiprazole and fluoxetine attenuated the increase in the BDNF-TrkB signaling in NAc as well as PFC, and hippocampus. Further detailed studies examining the underlying mechanism of action for the combination of brexpiprazole and fluoxetine in the NAc are needed.

Changes in dendritic length and spine density in the PFC and hippocampus are thought to contribute to the neurobiology of depression, and antidepressant treatment is mediated, in part, by blocking or reversing these changes (Duman and Aghajanian 2012; Ohgi et al. 2015; McEwen 2007). A single administration of TrkB agonist 7,8-DHF and TrkB antagonist ANA-12 could normalize alterations in spine density in inflammation model by stimulation at TrkB in the PFC, CA3, and DG, as well as blockade of TrkB in the NAc, respectively (Zhang et al. 2015a). Therefore, the combination of brexpiprazole and fluoxetine could act by normalizing altered dendritic spine density in all these regions, including PFC, hippocampus, and NAc. Thus, it seems that BDNF-TrkB signaling in NAc might play a role in the antidepressant effect of brexpiprazole plus fluoxetine, although further studies are needed.

Recently, Svensson et al. (2016) reported that brexpiprazole added to a SSRI escitalopram synergistically potentiated α-amino-3-hydroxy-5-methyl-4-isoxazolepropionic acid receptor (AMPAR) as well as *N*-methyl-D-aspartate receptor (NMDAR)-mediated neurotransmission and also electrically evoked excitatory postsynaptic potentials (EPSPs) in the rat mPFC. Furthermore, the NMDAR antagonist ketamine (10 mg/kg) enhanced AMPA and apparently to some extent also NMDAR-induced currents in the rat mPFC (Björkholm et al. 2015). Together, it is likely that rapid antidepressant effects of brexipiprazole and a SSRI may be related to ketamine’s antidepressant action.

In conclusion, this study shows that adjunction of brexpiprazole to fluoxetine can produce a rapid antidepressant effect in inflammation model of depression. Therefore, it is likely that adjunction of brexpiprazole to SSRI could produce a rapid antidepressant effect in patients with major depression.
